# Annotations

**Published:** 1901-06-08

**Authors:** 


### Annotations.

### Legal Restrictions on Quackery.

The withdrawal of the so-called Bell Bill, which was introduced into the Legislature of New York State with the object of controlling the operations of the various quacks who practise so freely among the citizens, and especially the citizenesses of that enlightened State, tends to show that in America, just as in England, public feeling is opposed to any attempt to make the profession of healing a close corporation. We have and we believe they have laws preventing persons from falsely assuming medical titles, but so far the freedom of the individual to place himself under the medical care of anyone he likes, whether quack or qualified, has not been interfered with. This is a condition which is often regarded by medical men as a scandal and a reproach to the intelligence of the country, and many of our profession heartily supported the general principle of the Bell Bill, which was to compel all the crowd of Christian Scientists, osteopaths, and quacks of various denominations to go through a medical education, which it was thought would greatly restrict their operations. But on the other hand it would have given all these good people a legal status, and take it altogether we are glad that the Bill was thrown out. From the professional point of view no doubt it would be a good and right thing that the quacks should be restrained. But it is out of the question to expect that any law-givers in any country will make it penal to give advice. The extreme to which any legislature is ever likely to go would be that it should demand, as did the Bell Bill, that every person who held himself out as able to advise on health matters should show that he had undergone a certain training and that he knew something about the machine which he was endeavouring to tinker. Any such proposal would, we fear, carry with it this serious consequence, that orthodox medicine would become but one sect among a whole crowd of healers all qualified before the law to practise their own hocus pocuses upon the public. Better let things alone.

### "Providence."

Those who regard thrift as among the greatest of the virtues not unnaturally look with favouring eye upon all schemes for providing beforehand, by small but frequently-repeated payments, for events which are sure sooner or later to happen, and are almost sure, when they do happen, to come, as the saying is, "all in a heap," and thus to involve ruinous expense. Nothing then from this point of view can be more appropriately paid for on the "provident system" than medical attendance; for while the weekly penny is a small affair, the doctor's bill is a serious business—would indeed sometimes be absolutely crushing, coming as it does just when sickness has tended to drain the family resources, had not the good people in too many cases discovered an easy way out of the difficulty by the simple plan of leaving it unpaid! For every reason, then, both for the doctors' and their patients' sake, the provident plan of payment would be ideal, if only some genius would discover a method of adjusting the payment to the services required. We need not here enter upon the endless difficulties with which the whole system has become entangled in England, difficulties largely due to the fact that the amount of the subscriptions was first fixed on a charity instead of a business basis; but we would point out that there are difficulties absolutely inherent in all such schemes, and that whether in England or on the Continent, whether in America or at the Antipodes, provident arrangements for the payment of doctors' bills have always led to friction and to dissatisfaction. As we have said the genius has not yet arisen to settle this apparently simple affair, and we are afraid he will not arise. It is not only, as some people think, a difficulty of price. It is a difficulty of quality; and as to this who is to judge? Quality of medical service can only be estimated by the public in accordance with the doctor's reputation, and directly a doctor gets a reputation for being better than his neighbours he throws up his clubs and provident arrangements and reaps his reward. Thus it happens, and always will happen, that the "best advice," as judged by the only criterion open to the public, can never be obtained under any "provident" scheme—and that is a little fact which the public had better put in its pocket to smoke at its leisure. All the same, if the said genius would kindly invent some acceptable scheme by which "providence" should be separated from charity, and by which well-to-do people should be enabled to ensure for medical attendance, not by sneaking into penny-a-week clubs set up for labourers and lower-grade artisans, but by paying a good substantial subscription for good substantial medical attendance, then, perhaps, would doctors' bills be things of the past, and the doctor's visits would be accepted without any *arrière-pensée* about the fee. Perhaps! But we are not sure that such a consummation would even then be possible. For though fashion may not rule in physic, it does undoubtedly in physicians, and whether it be a question of a doctor or a dancer whatever is the rage is pretty certain to cost money.

### Promotion by Merit.

One of the most difficult problems which the committee of experts to whom it is presumed the various proposals for the reorganisation of the Army Medical Service will be submitted will have to solve, will be that of combining such a degree of promotion by seniority as shall give to those who enter the service a reasonable assurance of advancement, with such a degree of promotion by merit as shall make it certain that, at least in appointing to the upper ranks, due consideration shall be given to professional efficiency. It is obvious enough that in the early stages of a medical officer's career, however brilliant in any particular field he may have shown himself as a student, he must go through the mill. He must serve on foreign stations, he must study disease and treatment in various climes, and he must prepare himself to undertake any of the multifarious duties which may at any moment fall to the lot of an officer of the Royal Army Medical Corps, and during this period of probation, which is really educational, seniority must rule. But what is essential is that every medical officer should feel that even from the beginning it is on the way in which his purely professional work is being done that his further promotion will depend, and that there is some reasonable probability that any special aptitudes which he may show himself to possess will be taken note of in deciding the sort of post that shall be offered to him. It is exactly in this direction that the existing system has mostly failed. Whatever else may be done with the system by which the R.A.M.C. is governed—and it is the system which is at fault—something must be done to reward professional merit, and by professional merit we mean something very different from neatness in filling up returns and readiness to conform to army traditions.

